# The Analysis of Autosomal STRs Draws the Current Genetic Map and Evolutionary History of Northernmost South America

**DOI:** 10.3390/genes16050574

**Published:** 2025-05-14

**Authors:** Julie Moncada Madero, Fernanda Mogollón Olivares, Dayana Suárez Medellín, Alejandra Coronel Guzmán, Andrea Casas-Vargas, William Usaquén Martínez

**Affiliations:** Grupo de Genética de Poblaciones e Identificación, Instituto de Genética—Universidad Nacional de Colombia, Sede Bogotá 111321, Colombia; jamoncadam@unal.edu.co (J.M.M.); mfmogollono@unal.edu.co (F.M.O.); ndsuarezm@unal.edu.co (D.S.M.); mcoronelg@unal.edu.co (A.C.G.); lacasasv@unal.edu.co (A.C.-V.)

**Keywords:** short-tandem repeats (STRs), human identification, human genetics, population genetics, anthropological genetics, genetic substructure analysis

## Abstract

Objectives: To analyze Colombia’s current human population, we employed a population genetics approach enriched by genealogical, demographic, cultural, and historical data to learn about its evolutionary history and to elucidate ethnic belonging and relationship patterns between its various population groups. Materials and Methods: This study relied on ten autosomal microsatellite markers (STRs) from 1364 individuals surveyed throughout the country. Aside from employing descriptive population genetics, substructure, and distance analysis, this investigation evaluated genealogical, demographic, cultural, and historical data gathered from fieldwork surveys. Results: We present a genetic diversity and ethnic belonging map of Colombia that suggests a nine-population classification (under Afro-descendant, Native American, and Admixed ethnicity labels) that reveals traces of evolutionary processes discussed in the light of the recent literature based on modern molecular markers. Colombia’s genetic trace from Africa varies among territories, as shown here by two differentiated Afro ancestral components, Chocó and San Andrés, in addition to the Afro admixture category. Some Native American peoples like the Wayúu, Zenú, Ticuna, Huitoto, and Cocama have a genetic configuration that remains relatively preserved. Nevertheless, other self-determined indigenous peoples who remain in their ancestral territories exhibit genetic introgression that is also reflected by their acculturation levels such as the Pijaos, Kankuamos, and Mokaná. The population classified as European admixture also shows an ancestral component that seems to be more fixed throughout neighboring territories but whose fluctuation depends on its specific demographic histories. Conclusions: This study combines STRs, a targeted sampling strategy, and advanced analytical tools to explore Colombia’s genetic diversity and evolutionary history. Locally, these findings enhance the understanding of genetics in a post-conflict society, crucial for human identification. Globally, they contribute to human population genetics, helping address evolutionary questions using data from diverse human ancestries and geographies.

## 1. Introduction

In the context of the Genetic Odyssey that was the peopling of the world [1], and as suggested by Cavalli Sforza [2], South America was the last subcontinent to be settled. Although humans entered the continent from Siberia through the Bering Strait, other migrations over time were equally relevant for the settlement of central and south America. Such journeys included those undertaken by Native Americans before and during vast pre-Hispanic empires, the arrival of Spanish conquerors, population movements during the development of the New World, and those driven by the wars of independence, the social reorganization of the 19th century and the great migrations of the 20th century.

Post-colonial migrations contributed to the shaping of the human populations of several Central and South American countries. Such is the case of the well-documented migrations from Italy to the south of the continent from 1870 to 1930 that arrived mainly in Brazil, Argentina, Chile, and Uruguay [3] and those from Germany, including Mennonite groups, that arrived in Brazil, Mexico, Paraguay, and Bolivia [4]. Population waves from East Asia, specifically from China and Japan, also arrived in some South American countries. In Peru, for example, there are reports of individuals with a proportion of East Asian ancestry above 25% that has been attributed to the arrival of Chinese workers during the second half of the 19th century [5]. Sometime later, and especially during the 1930s, Peru and Brazil received Japanese immigrants who came to the West in search of better economic prospects [6].

As in other Latin American countries, the patterns of genetic diversity currently observed in Colombia correlate with its history. During the settlement of South America, the Darien Gap was likely a mandatory passageway for the first settlers around 15,000 to 13,500 years ago [7]. Then, Native American communities established throughout the territory witnessed the arrival of conquerors from the old continent at the end of the 15th century. From then on, the native owners of the land would intermarry with those who arrived on conquistador and human trafficking ships to give rise to a tri-ethnic population that would live under the yoke of colonialism until achieving independence three centuries later [8]. In terms of migratory waves from outside the continent, Colombia was not the subject of genetic flow as much as other countries but received waves of Syrian, Lebanese, and Palestinian people in a sustained flow between 1890 and 1930 at its Caribbean coast [9].

In addition to this, the exploitation of cinchona bark and gums by local extractors in the Amazon started in the second half of the 19th century and gave rise to a solidly established rubber industry until the end of the century [10]. Aside from inspiring the writing of the novel *The Vortex*, this event changed the demography and admixture dynamics of this biogeographic region. During the first decades of the 20th century, “Mestizo” settlers started migrating from urban centers to less populated regions of Colombia in a spontaneous fashion or sponsored by the state [11]. Between 1946 and 1960, the civil war known as La Violencia (The Violence) caused the displacement of rural communities that would eventually give rise to peasant self-defenses in some regions throughout Colombia [12]. Since then, some populations have been constantly subjected to forced displacement by internal armed conflict in search of personal safety and job opportunities [13].

The history of human population movements through space and time is relevant when studying the genetic composition of the southern part of the continent, and a large amount of research about its peopling shows that it continues to be a fertile field of action. In the spirit of adding to the knowledge about the genetics of the population found at the gateway to the South American subcontinent, Colombia has sought to be at the forefront of technology and is currently focusing on the analysis of complete genomes [14,15].

On the other hand, genetic analyses of the Colombian population have traditionally revolved around political–administrative units, and place of birth has been used as the primary classification criterion. The Political Constitution of Colombia of 1991 established its current territorial political organization to establish the decentralization and autonomy of territories; however, the classification of human populations based on political criteria is far from ideal and results in artificial groupings. An alternative for the genetic study of human populations in Colombia implements, in addition to robust statistical and population genetic means, methodologies of anthropological genetics that consider different variables responsible for the distribution of the human species throughout the world, such as genetics, history, culture, and environment, among others [16].

In this work, we present a study of the current human population of Colombia based on 1364 samples obtained throughout the country. Here, we performed a population genetic analysis based on ten autosomal microsatellite markers (STRs) as informative molecular tools of evolutionary processes. We supported our genetic investigation with genealogical, demographic, cultural, and historical data to elucidate patterns of ethnic belonging and the existing relationships between the country’s various population groups. Finally, although our approach was not forensic, we report allele frequencies and commonly used forensic measures for each population group considered.

## 2. Materials and Methods

### 2.1. Sample Selection and Classification

This study used samples collected throughout the country between 2008 and 2017 by the Population Genetics and Identification research group of the Universidad Nacional de Colombia. Sampling was performed in association with local medical centers, and inclusion criteria included adults above 18 years old who were born in the sampling area or resided there for at least two years. Each participant had informed consent and information from a survey that considered genealogical, demographic, and socio-cultural variables.

**Figure 1:**
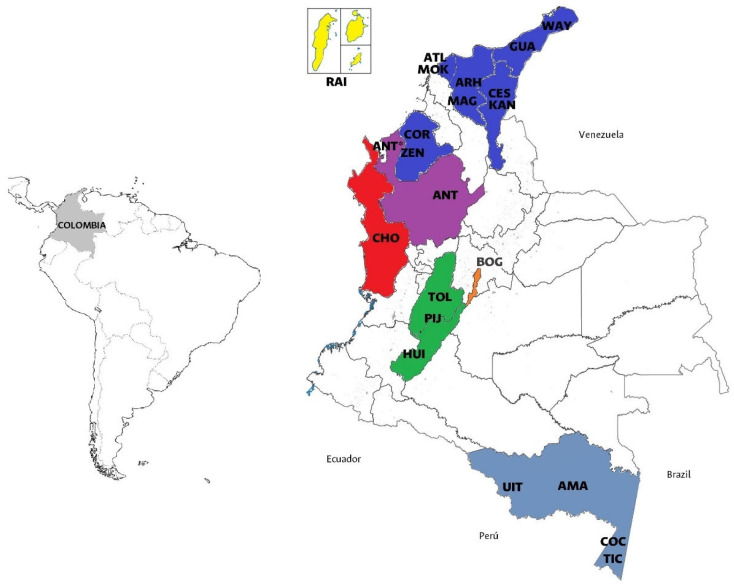
Geographic distribution of populations analyzed. Caribbean, Insular, and Pacific populations are depicted in dark blue, yellow, and red, respectively. Amazonian populations are depicted in dark gray. Andean populations such as Tolima (TOL), Pijao (PIJ), and Huila (HUI) are depicted in green and Bogotá (BOG) is in orange. Antioquia is depicted in purple.

Table 1 shows the a priori genealogical classifications based on the collected information. Although some categories were named after the departments, they were not limited to the political–administrative context since they were based not only on birthplace but also on the self-identification of three generations of each participant’s paternal and maternal lineages. Overall, 364 unrelated individuals were grouped in 24 a priori genealogical classifications. Figure 1 shows their geographic distribution.

### 2.2. DNA Extraction, Amplification, and Genotyping

Samples were processed at the Population Genetics and Identification laboratory of the Universidad Nacional de Colombia. Peripheral blood and/or buccal mucosal epithelial cells were collected using FTA™ WHATMAN^®^ cards, and DNA extraction was performed with Chelex [17] FTA Whatman^®^ buffer and FTA Elute Whatman^®^ protocols. Multiplex PCR amplification was completed using two different kits on different sample sets: 15 autosomal STR loci with the AmpliF/STR Identifiler kit (Applied Biosystems, Warrington, UK); and 16 autosomal STR loci with the AmpFLSTR^TM^ NGM^TM^ PCR Amplification Kit (ThermoFisher, Waltham, MA, USA) (the Identifiler and the NGM kits have the same primers in the common markers). Capillary electrophoresis was run on an ABI Prism 310 Genetic Analyzer (Applied Biosystems, Stafford, TX, USA). Data were processed and genotyped using Genemapper ID v.3.2 software (Life Technologies, Waltham, MA, USA). The samples in this study were collected over more than ten years of research, so they were processed at different times using distinct marker kits. The analysis presented here employed the ten common markers among all data sets: D16S539, D18S51, D19S433, D21S11, D2S1338, D3S1358, D8S1179, FGA, TH01, and vWA; these were included in the analysis.

### 2.3. Statistical and Population Genetic Analysis

Population genetic analysis was based on the genealogical classifications described above. Allele frequencies, effective alleles, the Hardy–Weinberg equilibrium, and observed and expected heterozygosities [18] were calculated using Genepop v 4.6 [19]. The Arlequin v. 3.5.2.2 software [20] was also used for calculating the Hardy–Weinberg equilibrium, as well as for the analysis of molecular variance (AMOVA) and to perform paired Fst analyses with 10,000 permutations and a significance level of 0.05. A NeighborNet based on the paired Fst matrix was constructed using the Splits Tree4 v. 4.15.1 software [21] to graphically represent the relationships between populations.

Subsequently, the Structure 2.3.4 software [22] provided an individual population assignment analysis under the admixture model and correlated allele frequencies with a burning length of 100,000 and 100,000 MCMC repeats. Five independent simulations were performed for k = 2–10 to obtain the most likely k by determining the modal distribution of ΔK [23] using Structure Harvester [24] to find the number of gene clusters (K) that best fit these data. The results were uploaded to the Clumpak—Cluster Markov Packager Across K software for visualization.

The neighbor joining clustering methodology [18] was used to evaluate genetic distances using the Populations 1.2.31 software and TreeView X to plot the results. A multivariate analysis was also performed. Using the default parameters of the MVSP v.3.22 software [25], principal component analysis was obtained. Finally, a Mantel test was run to analyze the relationship between geographic and genetic distances and to evaluate independence between both matrices and spatial autocorrelation. This last test used the mantel.test function from the free R development [26], employing 10,000 permutations and an α = 0.05.

### 2.4. Classifications Based on Ethnic Belonging

Genetic diversity and Hardy–Weinberg equilibrium analyses were also performed for the final ethnic classifications suggested by this study, and forensically relevant statistics were calculated using the PowerStats v12.xls tool. Additionally, such classifications were compared to populations from different geographies published by other authors (Table 2). This data set was composed of various ancestral populations. We included samples from Native American and admixed people from Central and South America, as well as from East and West Africa and Europe. We performed principal component analyses using the MVSP 3.22 software [25]. The PCA analyses included data from all populations, as well as independent analyses of those with mainly African and Native American ancestry.

## 3. Results

### 3.1. A Priori Classifications Based on Genealogy

**Figure 2:**
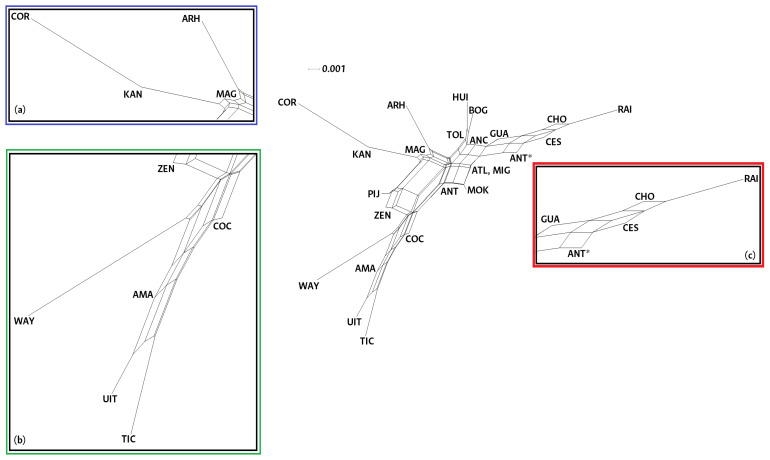
A NeighborNet constructed from the paired Fst values of the 24 populations studied. At the center of the graph, there are multiple ancestral populations (ATL, ANT, HUI, BOG, ANC, TOL, and MIG). (**a**) Native American and admixed communities from the Caribbean coast (KAN, ARH, COR, and MAG) (blue). (**b**) Indigenous communities (ITU, TIC, AMA, COC, WAY, and ZEN) (green). (**c**) Afro-descendant populations (RAI, CHO, ANT*, CES, and GUA) (red).

The NeighborNet in Figure 2 presents the paired Fst results. This representation shows populations that aggregate according to ethnicity and geographic location. There are three distinct regions: Native American, admixed, and Afro. There is one extra region associating people with various genetic and cultural histories. On the right side of the graph, there are the Colombian Afro-descendant communities of Chocó, the Raizales of San Andrés and Providencia, and the Afro-descendant populations of Guajira, Cesar, and Antioquia. The upper part of the net shows the most admixed populations of our sample: those in the Migrants, Multiple ancestries, Magdalena, Tolima, Atlántico, Antioquia, Huila, and Bogotá classifications. The bottom left part of the NeighborNet groups the Cocama, Ticuna, and Uitoto Amazonian indigenous communities near the Amazonian population, while the Wayúu are in an independent branch next to the Zenú communities. Finally, at the top left of the net, we find the Arhuacos and Kankuamos (Native American communities from the Caribbean coastal mountains) as well as the admixed people from Cordoba and Magdalena (also from the Caribbean coast). Despite being related to the Arhuacos and the Kankuamos, the Mokaná people appear in the mid-section of this graph. The Pijaos, self-determined acculturated natives from the Andean region, are in the middle of the two main Native American descent groupings. A neighbor joining dendrogram constructed based on the pairwise Fst values from the 24 populations studied show similar groupings (Figure S1).

**Figure 3:**
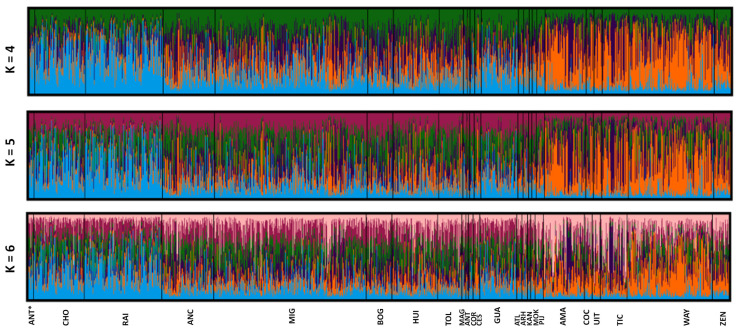
Bar diagrams showing ethnic belonging inferred from individual assignment (k = 4; k = 5; and k = 6), with k = 5 being the best fit to the data (Figure S8).

The a priori genealogical categories were also analyzed by individual assignment to the populations in which simulations were performed for K = 2–10 (Figure S2). According to the method of Evanno et al. (2005) [23], the number of genetic groups (K) that best explained these data was K = 5 (Figure 3). The bar charts obtained once again show what appear to be three groups with different ancestral prevalences. At k = 5, the Afro-descendant (ANT*, CHO, and RAI) and the Guajira populations show assigned individuals represented by a mostly blue genetic component. In the middle band of the graph, multiple-ancestry populations (ANC, MIG, BOG, HUI, TOL, ANT, MAG, and CES) together with the Arhuacos, Kankuamos, Mokaná, and Pijaos exhibit individual bars that suggest a higher degree of admixture. Finally, from the Amazonians to the Zenú, a pattern dominated by the orange color which we relate to Native American ancestry is observed. In the case of the Córdoba population, a predominantly orange pattern is also evident, which coincides with their grouping with the native populations in Figure 2.

The results from the Nei genetic distance analysis employing the neighbor joining clustering methodology support the groupings suggested by previous tests (Figure S3). Likewise, this dendrogram confirms some previously observed proximities, such as those between the Guajira population and the Afro communities, between the Córdoba and the Zenú, and between the native communities from the Caribbean coastal mountains (KAN, ARH, and MOK) and the admixed populations from the departments they inhabit (MAG and ATL). Similarly, the principal component analysis shows that the clouds of data points scattered along the axes aggregate according to their ethnic belonging (Figure S4).

The Mantel test, a method to assess the relationship between geographic distance and genetic divergence, was used to evaluate the similarity between those two matrices (Figure S5). In this data set, we found a statistically significant correlation between genetic divergence (Fst) and geographic distances (km) (*p*-value < 0.0001; α = 0.05; r(AB) = 0.387) that suggests an adherence to the isolation-by-distance model. However, considering their correlation coefficient, these results should be interpreted with caution and suggest the need for further research.

### 3.2. Final Population Classifications

After having performed population genetics and statistical analysis based on the original classifications, we suggest a new classification that more closely reflects the genetic structure of the Colombian population. Table 3 shows the nine new classifications under the labels of Afro-descendant, Native American, and Admixed ethnicity.

### 3.3. Genetic Diversity Analysis

Given the importance of considering our country’s genetic structure in forensic practice and hypothesis formulation in future population genetics studies, here, we report the allele frequencies (Table S1) and forensically relevant statistics of the nine final classifications (Table S2). Table S3 shows the observed heterozygosity (Ho) and inbreeding coefficient (Fis) values for each of the nine final genealogical classifications. The Wayúu had the lowest observed heterozygosity at 0.756 and the Afro admixture classification the highest one at 0.837. These results show a correlation between genetic and geographic distances, following the model that suggests heterozygosity losses throughout a series of founder effects experienced by the human population as it moved away from East Africa [33].

Regarding the Hardy–Weinberg equilibrium, there were some loci scattered throughout the population categories that exhibited *p*-values below 0.05. There was not a clear pattern of disequilibrium in a single locus throughout the population categories studied, and observed imbalances were solved after adjusting the *p*-values using the Benjamani–Hochberg method, except for the D19S433 marker of the Native American admixture group (Table S4).

### 3.4. Genetic Structure Analysis

We evaluated the genetic structure of the whole sample using a four-group AMOVA to test for differentiation levels. The general population exhibited variance mostly among individuals (97.63%) with a *p*-value < 0.05 (Table S5). Additionally, paired Fst matrices showed no significant genetic differences between the proposed genealogical categories (Table S6).

### 3.5. Comparative Analysis

**Figure 4:**
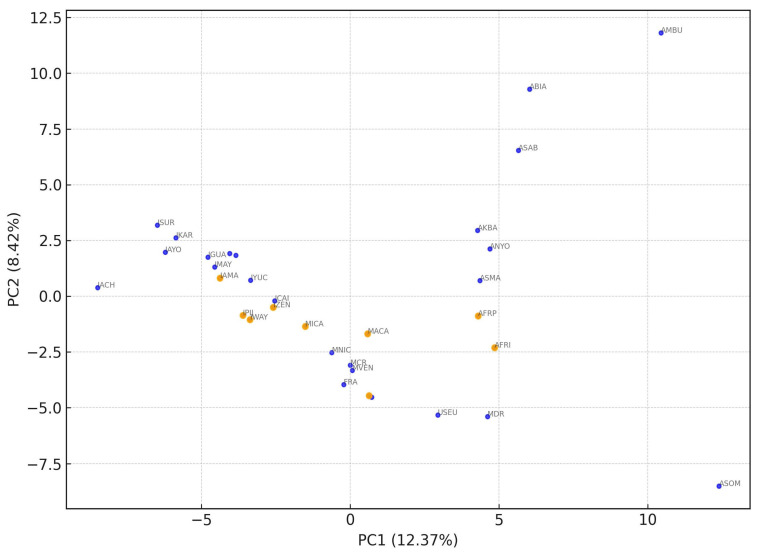
A two-dimensional plot of the principal component analysis from this study and comparison populations as well as eigenvalues: Component 1 (12.37%) and Component 2 (8.42%).

Figure 4 shows a comparison between our nine classifications (orange markers) and other populations (blue markers) using a principal component analysis. The two-dimensional representation of the results shows a dispersion along the two axes according to African, European, and indigenous ethnicity, respectively, from left to right. Afro populations from Colombia and the admixed population from the Dominican Republic lie between African and European populations, and the admixed Latin American populations aggregate in the center of the graph, while the indigenous ones disperse along the two quadrants on the right.

We performed separate PCAs to assess closeness between populations with African and indigenous ancestry (Figure S6 and Figure S7, respectively). These results show that the Afro-Pacific sample is closer to the admixed Dominican and the admixed Afro-Colombian samples, while the Afro-Insular sample is closer to the Mandinga people from Senegal. The indigenous populations, on the other hand, are scattered through the graph according to their geographic location. The Caribbean admixed indigenous category is at one end of the dispersion.

## 4. Discussion

The differentiation of Colombia’s current human population goes beyond any artificial grouping based on its political–administrative units. The results presented here rest on anthropological genetics methodologies and autosomal STR markers that expose diverse patterns of ethnic belonging and relationships between groupings that allude to shared evolutionary histories.

STR markers are large polymorphisms originally used for assessing the mechanisms of evolution in population genetics analyses. Before them, other markers sought to unravel evolutionary signals, such as the HLAs that detect genetic drift through polymorphism-level differences between generations and populations, along with the ABO blood system that acts as a clear natural selection marker [34]. An example of how informative these classical markers can be is that regarding Amerindian communities who used to carry only the O allele, it is currently possible to evaluate more than 500 years of gene introgression by calculating the frequencies of the O, A, and B alleles to infer the changes they have undergone since conquest.

Amid a technological race that constantly seeks to employ newer markers and the possibility of exploring the genomics of a population, it should be noted that classical markers continue to be informative of evolutionary processes that answer questions arising in the field of population genetics. Undoubtedly, complete genomes allow for exhaustive analyses of populations, but these technologies are not yet easily accessible to many Latin American research groups that, however, do have access to valuable samples for ancestry studies. Also, there is a correlation between genetic ancestry estimations obtained with ancestry-informative markers (AIMs) and high-density data [35]. Such correlation implies that the use of new technologies does not exclude the potential of analyses performed with markers whose databases are freely accessible and contain a robust accumulation of information over time, like the sample analyzed in this study. STRs can stratify populations at the continental level, and whenever accompanied by a body of complementary a priori information from the research subjects, they can also be good at performing so at a local scale.

Several works published about Colombia’s population ancestry have used the Native American, Afro-descendant, and European ancestry categories as if they were immoveable. However, our results show that considering that such categories respond to demographic history, their nature is more dynamic. Here, we started with an a priori classification of Colombian population samples representative of several of its regions (Table 1). We proposed such classifications considering personal, genealogical, demographic, and cultural information. However, once we obtained the substructure and ethnic belonging assignment results (Figure 2 and Figure 3), we suggested a reclassification that includes two Afro-type populations, three Native American-type populations, and four admixed-type populations (Table 2). Similar groupings were previously proposed based on AIMs [36,37].

The history of the American continent provides a general idea of how the gene pool of human populations reflects their subjection to mechanisms of evolutionary change over time. The settlement of European immigrants, for example, used to coincide with epicenters of the indigenous civilizations they encountered during their colonizing missions, while the settlement of African populations coincided with centers of slave trading and labor demand. Likewise, after the early 19th century’s independence wars, the degree of admixture in the New World depended on the territory, the degree of migration, and the legislation of each region [35].

Similarly, Colombia’s current population reflects the historical dynamics of indigenous settlements established before the colony, the arrival of Africans from different geographies in different regions and times, and the subsequent admixture of these two groups with the European population. The genetic trace from Africa varies among territories, as shown here by two differentiated Afro ancestral components, Chocó and San Andrés, in addition to the Afro admixture category. We also found that some Native American peoples like the Wayúu, Zenú, Ticuna, Huitoto, and Cocama have a genetic configuration that remains relatively preserved. Nevertheless, other self-determined indigenous peoples who remain in their ancestral territories exhibit genetic introgression that is also reflected by their acculturation levels (loss of language) such as the case of the Pijaos, Kankuamos, and Mokaná. Finally, the population classified as European admixture also shows an ancestral component that seems to be more fixed throughout neighboring territories but whose fluctuation depends on their specific demographic histories.

As mentioned above, microsatellite markers are not ancestry-informative markers but make it possible to trace evolutionary change at a more recent scale. As our results show, STRs can be used to assign individuals to specific populations and ethnic belongings. The groupings obtained reflect similar origins, migrations, and drift histories, maybe not dating as far back as to reveal their ancestral history, but one that arose at a more recent time scale.

The network presented in Figure 2 shows people from Córdoba and Amazonas who did not self-determine as indigenous aggregating to the Native American branches from such regions; this is a reflection of their shared geography that does not imply shared ancestry but recent admixture. It is worth highlighting that these samples come from field sampling at locations with a high degree of ethnic belonging, as reported by the National Administrative Department of Statistics (DANE). Likewise, the close association between the Guajira, Cesar, and Afro branches does not imply African descent but might result from the introgression of African genomes in the admixed population of the region. A history review unveils migrations and genetic flow caused by the exploitation of pearl banks on the Guajira coast during the 16th century that required an enslaved labor force for agriculture, farming, and housework and as fishermen diving for pearls who were brought to the country from Guinea and Angola [38].

The Native American admixture category came up given the reiterated association between native populations from the Caribbean coastal mountains and admixed people from Magdalena and Atlántico. As discussed above, these samples were collected at a location with a high degree of ethnic belonging, so even when their genealogical data and self-determination initially suggested independent categories, there is evidence of gene flow in their genomes. In contrast, despite having a small sample size from the native communities from the mountains (Arhuacos, Kankuamos, and Mokaná), SNP AIMs analyses have shown that their tendency to associate does stem from a shared ancestry and consequent gene flow [39].

The landscape of peopling processes and gene flow at the gateway of South America extends throughout the subcontinent. Based on SNP data from South America, Homburger et al., 2015 [5], found substructure in populations of European ancestry, depending on whether they came from northern or southern Europe, as well as in those of Native American ancestry. According to the authors, in Peru, there is substructure between the local Andean Quechua and Aymara native groups, while the genomes of indigenous peoples from Argentina and Chile carry a component from the south-center of the subcontinent and another one from the Andes.

The matter of multiple origins of indigenous ancestry in South America has been addressed over time using several genetic markers. The results from this study show a separation between the indigenous communities of the Caribbean and the Amazon (Figure 2). Previous local studies have discussed the genetic differences between Amerindian groups in the Amazon and Orinoco regions compared to those from the Andes and the Caribbean coast [40]. Using autosomal STR markers, a difference in genetic diversity from west to east between Andean and eastern Brazilian tribes was reported as one of the strongest signals of subcontinental genetic differentiation [41]. Analyses of parental lineages have also reached conclusions about this issue. In terms of the Y chromosome, there is a significant geographic structure of genetic variability between the Andean and western populations caused by mechanisms of evolution acting differently throughout South America [42]. The differential evolutionary model that causes a contrast between east and west was later upheld by analyses based on mitochondrial DNA [43].

Several theories about the settlement of the southern part of the continent have attempted to account for the existence of different types of indigenous ancestry. It has been suggested that the first settlers could have entered in a single wave that later separated into three migratory routes that would reach the Andes, the Amazon, and the coastal regions [7,44] or in two independent waves that entered from each side of the Andes, based on the observation that some Amazonian ethnic groups show more similarities with Australasian populations than with Eurasians or current Native Americans [45]. A later study suggested that the signal linking Native Americans from the Brazilian Amazon with “Australasians” may have been the result of a substructure within Siberian populations that gave rise to Native American ancestors [46].

Although they do not exhibit as strong of a separation as the two distinct types of indigenous ancestry in our sample, we found differences in the association grouping between the populations of African descent from mainland Chocó and the island of San Andres. The PCA results that compare our samples with other African or African-descended populations (Figure S5) show that the Afro-Pacific samples are closer to the admixed Dominican Republic and the admixed Afro-Colombian population, while the Afro-Insular population is closer to the Mandinga from Senegal.

Also, studies based on uniparental markers, such as Y chromosome analyses, reveal a higher proportion of the haplogroup E1b1a among the Colombian population [47,48,49,50]. Population analyses from Chocó, Bolívar, and Valle del Cauca have reported the presence of such a haplogroup in frequencies of around 30% [48] while in populations from San Andrés and Providencia, its frequency is 48% [50]. The haplogroup E1b1a reaches its highest frequencies in western, central, eastern, and southern regions of Africa [51], which is consistent with the fact that the ancestors of the Afro-Colombian populations were brought and enslaved between 1580 and 1650 from Senegal, Ivory Coast, Mali, and the west coast of Guinea [52].

Ultimately, the genetic map of Colombia presented here exhibits a correlation between ethnic belongings and geography, as is the case in several Central and South American countries. Whole-genome analyses show that ancestral differences among admixed Latin Americans reflect, still today, the genetic imprint of the diversification underwent by pre-Columbian indigenous populations [53]. Other studies that have employed haplotype-based methods show significant geographic correspondence between native ancestral components in Latin America and the genetic structure of current native groups [5,31]. For example, the genomes of today’s admixed Mexicans reflect a high degree of fine-scale genomic structure shaped by pre-Columbian population dynamics [54]. According to researchers, such a continuous geographic distribution of each indigenous component demonstrates a high correlation of individual admixture proportions with geography. In Colombia, SNP-based research has shown that people from the northwest of the country carry ancestral components resembling those of Chibchan–Paezan natives and Central American Mayans, while to the south, there is an ancestral resemblance with Peru and Chile that is consistent with the central Andes being highly populated during the peak of the Inca Empire [55]. Finally, based on whole-genome data, it has been shown that Native Americans from Peru exhibit distinct ancestral divisions and that admixed populations have arisen from multiple indigenous communities that occurred before and during the Inca Empire and Spanish rule [56]. The authors of this study also point to a strong biogeographic signal within the genetic variation in indigenous and admixed populations. 

## 5. Conclusions

In the search for unraveling our country’s genetic diversity and evolutionary history, the combined use of STRs, a preconceived sampling strategy aimed at collecting personal, genealogical, demographic, and biological data, as well as robust statistical and population genetics analytical tools, led to notable findings. We present a genetic diversity and ethnic belonging map of Colombia that suggests nine classifications of its populations and reveals traces of evolutionary processes discussed in the light of the recent literature based on modern molecular markers. Such classifications indicate a correlation between ancestry and geography and, given their dynamism, emphasize the need to change obsolete colonial labels such as Caucasian or Mestizo. They also lead us to acknowledge the added value that researchers’ deep knowledge of their study populations provides for obtaining additional layers of information.

The results presented here do not intend to declare STRs as the best alternative for this type of research. They put forward several population classifications supported by the extra information layers provided by anthropological genetics, classifications comparable to those suggested by modern molecular markers. On a local scale, these findings contribute to a deeper understanding of the genetics of a country going through a post-conflict era with an increasingly pressing demand for the human identification of its victims. On a more global scale, they contribute to the field of human population genetics when breakthroughs in molecular biology make it possible to answer increasingly concrete evolutionary questions that require information representative of the genetic variability of human populations from all ancestries and geographies.

## Figures and Tables

**Table 1:** A priori classifications based on genealogical and birthplace information.

Classification	Abbreviation	n	Criteria
Bogotá	BOG	50	Participants born in Bogotá whose data were obtained from filiation cases. They reported the highest paternity rates, and both their parents were also born in Bogotá.
Tolima	TOL	47	Participants who did not report belonging to any ethnic group and whose parents were also born in the given department.
Huila	HUI	89	
Amazonas	AMA	80	
Atlántico	ATL	10	
Antioquia	ANT	8	
Cesar	CES	9	
Córdoba	COR	13	
Guajira	GUA	72	
Magdalena	MAG	5	
Afro-Antioquia	ANT*	10	Participants from Antioquia who were born in the municipalities of Vigía del Fuerte, Necoclí, and Turbo who self-determined as Afro-descendants.
Chocó	CHO	99	Afro-descendants from Chocó.
San Andrés and Providencia	RAI	151	A total of 117 Raizales and 34 born there whose mother and father are also from San Andrés and Providencia
Wayúu	WAY	166	Participants who self-determined as belonging to a specific Native American ethnicity.
Zenú	ZEN	32	
Arhuacos	ARH	11	
Kankuamos	KAN	6	
Mokaná	MOK	9	
Pijao	PIJ	16	
Ticuna	TIC	52	
Uitoto	UIT	16	
Cocama	COC	15	
Migrants	MIG	297	Participants whose parents were born in a different region from themselves.
Multiple ancestries	ANC	101	Individuals whose paternal and maternal lineages differed from each other.

**Table 2:** Populations used for the comparative analysis.

Population	Location	Code	Reference
Maya	Mexico—Native	IMAY	pop.STR—USC *
Pima	Mexico—Native	IPIM	pop.STR—USC
Tepehua	Mexico—Native	ITAP	[27]
Yucatán	Mexico—Native	IYUC	[28]
Caingang	Brazil—Native	ICAI	[29]
Guarani	Brazil—Native	IGUA	[29]
Karitiana	—	IKAR	pop.STR—USC
Surui	Brazil—Native	ISUR	pop.STR—USC
Aché	Paraguay—Native	IACH	[29]
Ayoreo	Paraguay—Native	IAYO	[29]
Venezuela	Venezuela—Admixed	MVEN	[30]
Costa Rica	Costa Rica—Admixed	MCR	[31]
Nicaragua	Nicaragua—Admixed	MNIC	[32]
Dominican Republic	Dominican Republic—Admixed	MDR	pop.STR—USC
Biaka Pygmies	Central African Republic	ABIA	pop.STR—USC
Mbuti Pygmies	Democratic Republic of Congo	AMBU	pop.STR—USC
Bantu N.E.	Kenya	AKBA	pop.STR—USC
Yoruba	Nigeria	ANYO	pop.STR—USC
Mandenka	Senegal	ASMA	pop.STR—USC
Somalia	Somalia	ASOM	pop.STR—USC
Bantu	South Africa	ASAB	pop.STR—USC
U.S. Europeans	United States	USEU	pop.STR—USC
French	France	FRA	pop.STR—USC
N.W. Spain	Spain	SPN	pop.STR—USC

* Database of the University of Santiago de Compostela, http://spsmart.cesga.es/popstr.php (accessed on 24 November 2024).

**Table 3:** Classifications of Colombian population according to ethnic belonging.

	Agrupación	Code	Participantes
AFRO	Afro-Insular	AFRI	Raizales
	Afro-Pacific	AFRP	Chocó and Antioquia Afro
NATIVE AMERICAN	Wayúu	IWAY	Wayúu
	Zenú	IZEN	Zenú and Córdoba
	Native Americans from the Amazon	IAMA	Ticuna, Uitoto, Cocama, and Amazonas
ADMIXED	Pijaos	IPIJ	Pijaos
	Native American admixture	MICA	Kankuamos, Arhuacos, Magdalena, Mokaná, and Atlántico
	Afro admixture	MACA	Guajira and Cesar
	European admixture	MEU	Tolima, Huila, Bogotá, Antioquia, Multiple ancestry, and Migrants

## Data Availability

All data generated or analyzed during this study are included in this published article. Protocols and deidentified, aggregated data that underlie the results reported in this article are available for non-commercial scientific purposes upon reasonable request from the corresponding author. For privacy reasons, raw data are not publicly available.

## Supplementary Materials

The following supporting information can be downloaded at: https://www.mdpi.com/article/10.3390/genes16050574/s1, Figure S1: A neighbor joining dendrogram constructed based on the pairwise Fst values of the 24 populations. Figure S2: Bar diagrams showing ethnic belonging inferred from the individual assignment (K = 2–10). Figure S3: A neighbor joining dendrogram constructed based on the results from the Nei genetic distance analysis of the 24 populations. Figure S4: A two-dimensional plot of the principal component analysis from the 24 study populations. Figure S5: The Mantel test. A. A summary graph. B. A histogram. The *p*-value was calculated by employing the distribution of r(AB) estimated from 10,000 permutations (*p*-value < 0.0001; α = 0.05; r(AB) = 0.387). Figure S6: A two-dimensional plot of the principal component analysis to assess closeness between populations with African ancestry. Figure S7: A two-dimensional plot of the principal component analysis to assess closeness between populations with indigenous ancestry. Figure S8: k = 5 was the best fit, based in delta K. Table S1: Allele frequencies for the 10 STR markers in each of the 9 new categories obtained from the previous 24 classifications. Table S2: Forensically relevant statistics for the 10 STR markers in each of the 9 new categories obtained from the previous 24 classifications. Table S3: Hardy–Weinberg equilibrium, expected and observed heterozygosities, and Fis indices per locus and per population. Table S4: Hardy–Weinberg’s significance level implementing Benjamani–Hochberg’s adjustment. Table S5: AMOVA calculated for the population categories analyzed in the present study. Table S6: Pairwise Fst values are shown below the diagonal. The *p*-values are shown above the diagonal. (**) represents the estimated Fst that were significant (1000 permutations; *p* < 0.05) for the 9 populations analyzed and the bold values show *p*-values > 0.05.
