# Supplementary figures and images for: The Analysis of Autosomal STRs Draws the Current Genetic Map and Evolutionary History of Northernmost South America
Source: Genes (Basel). 2025 May 14;16(5):574. doi: 10.3390/genes16050574 (PMC12110986; doi:10.3390/genes16050574)

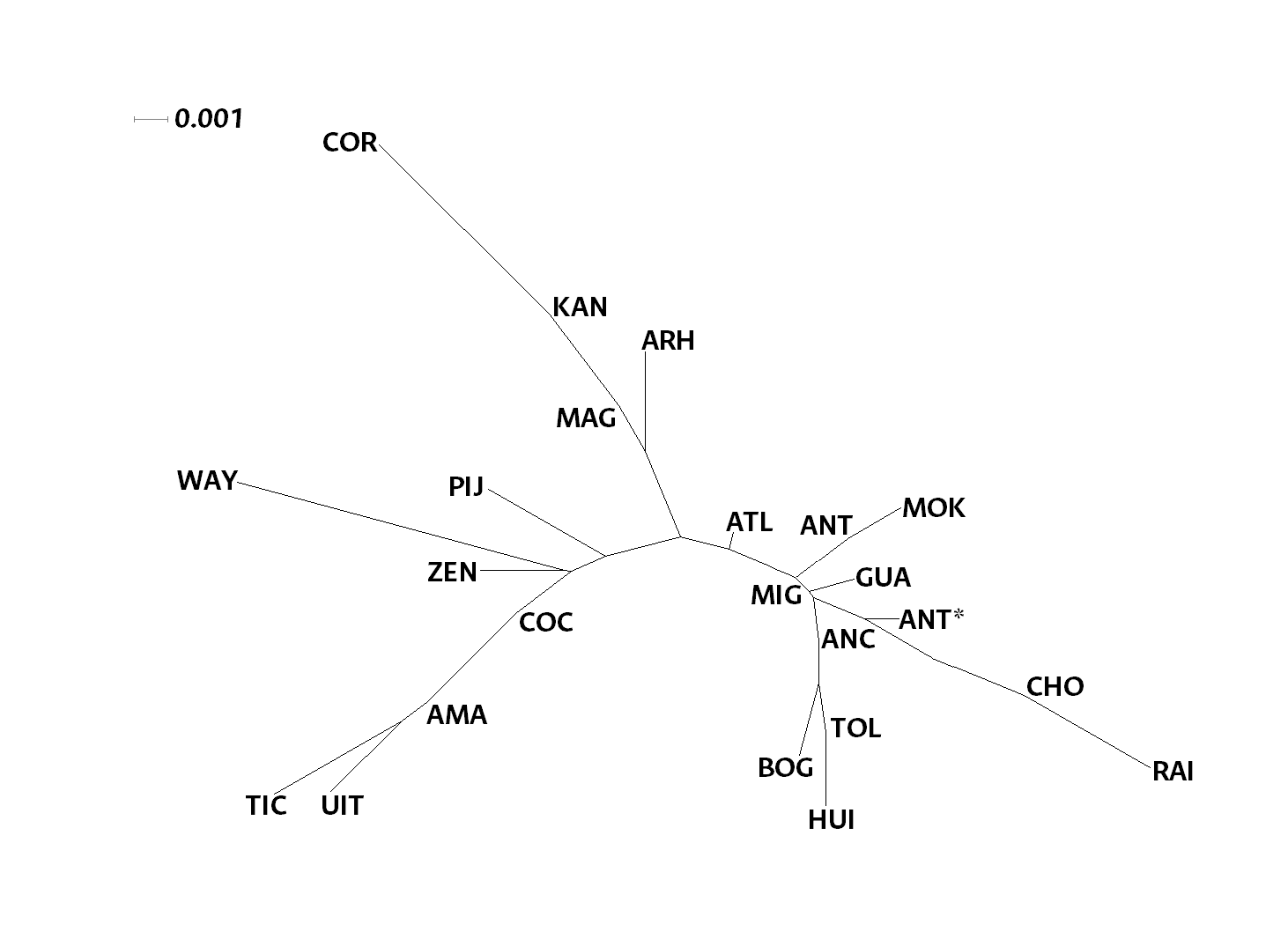

Supplement: Supplementary file 1 [file genes-16-00574-s001.zip › Figure_S1_SuppInfo.png]

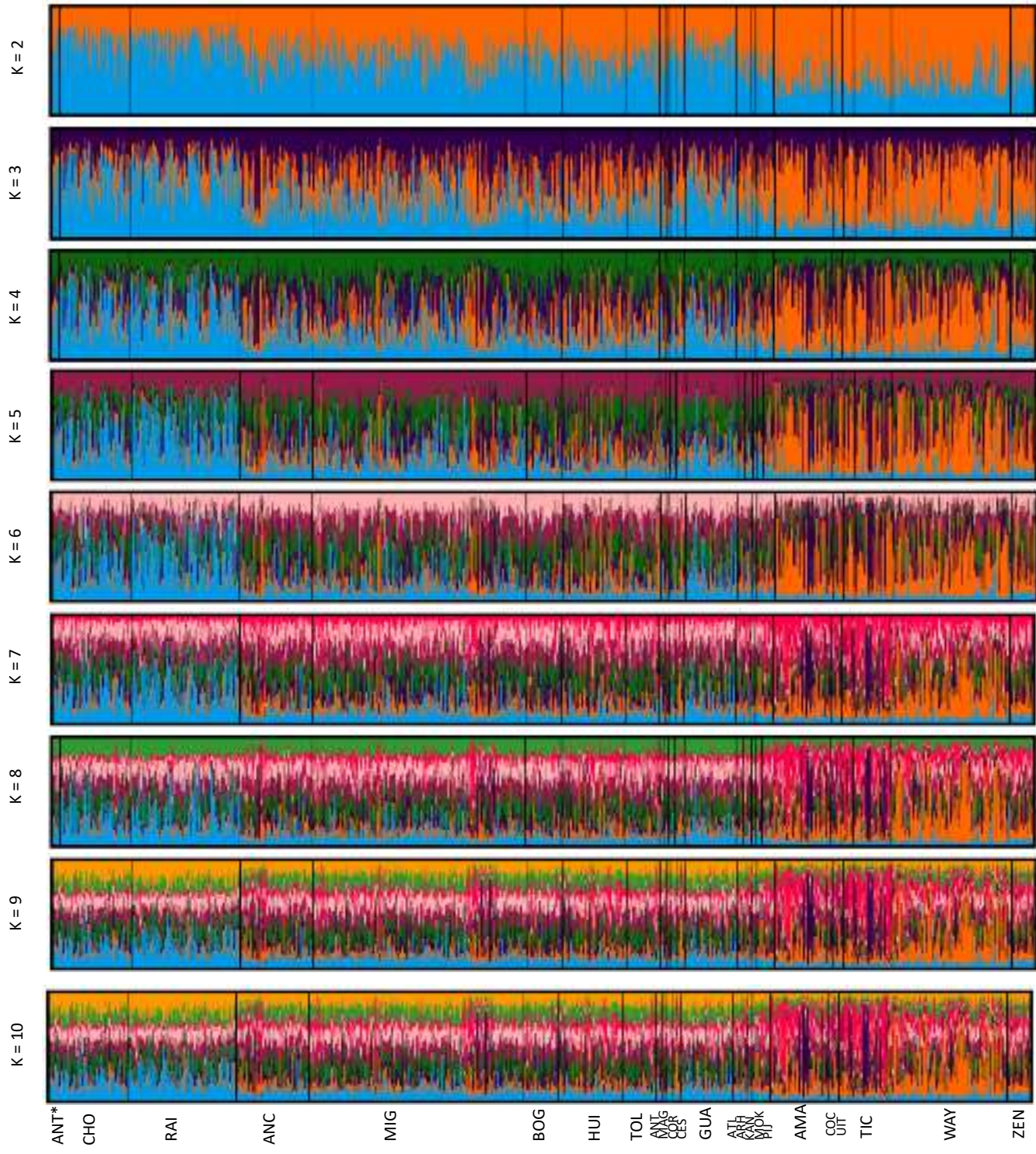

Supplement: Supplementary file 1 [file genes-16-00574-s001.zip › Figure_S2_SuppInfo.pdf]

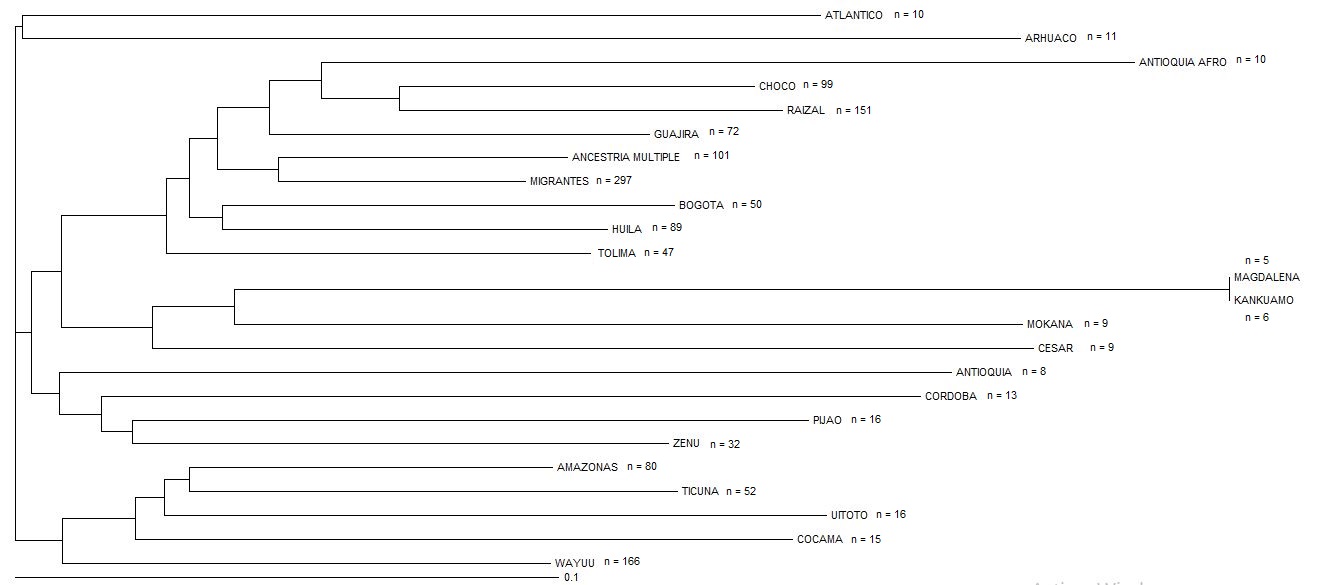

Supplement: Supplementary file 1 [file genes-16-00574-s001.zip › Figure_S3_SuppInfo.JPG]

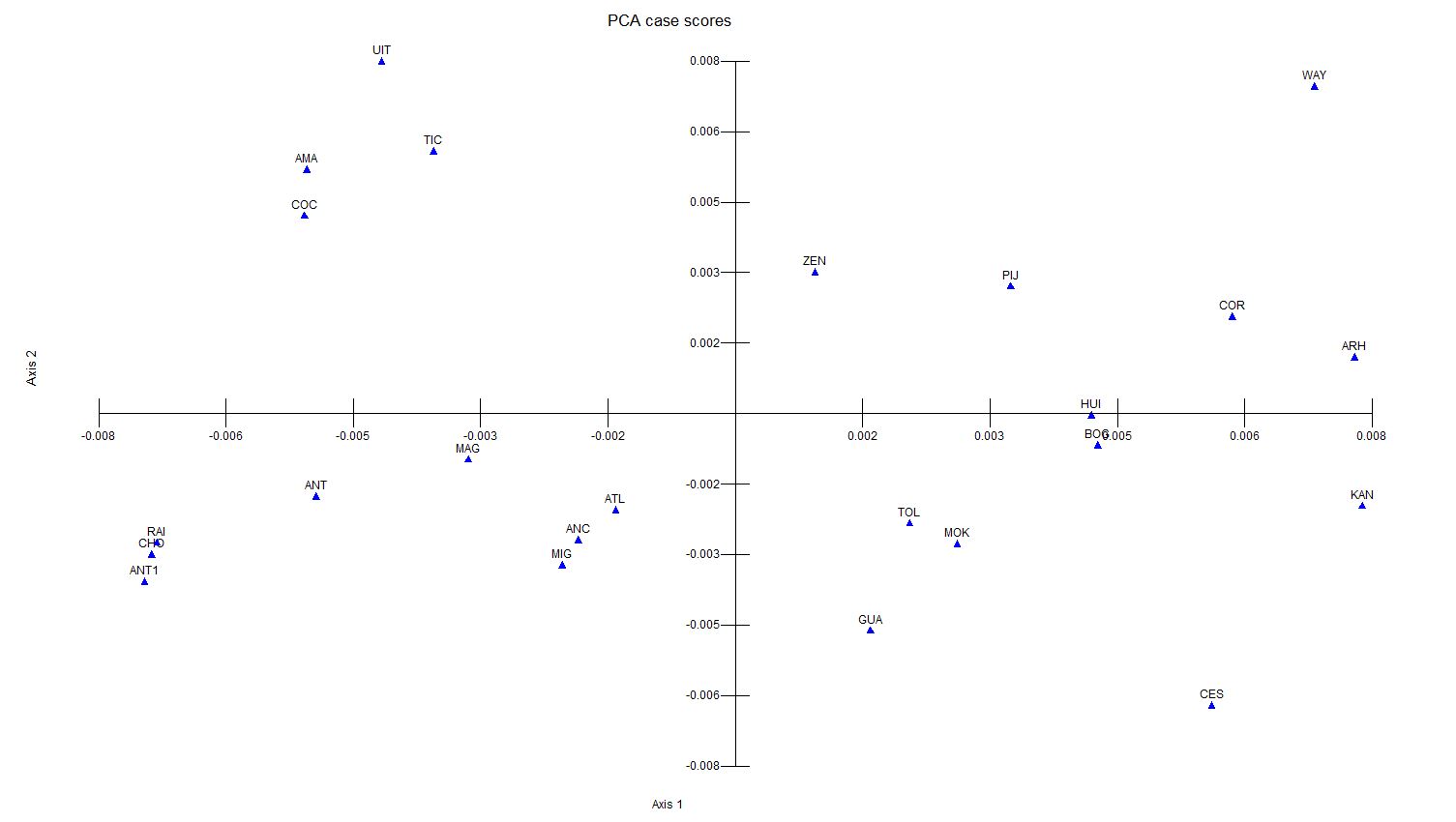

Supplement: Supplementary file 1 [file genes-16-00574-s001.zip › Figure_S4_SuppInfo.JPG]

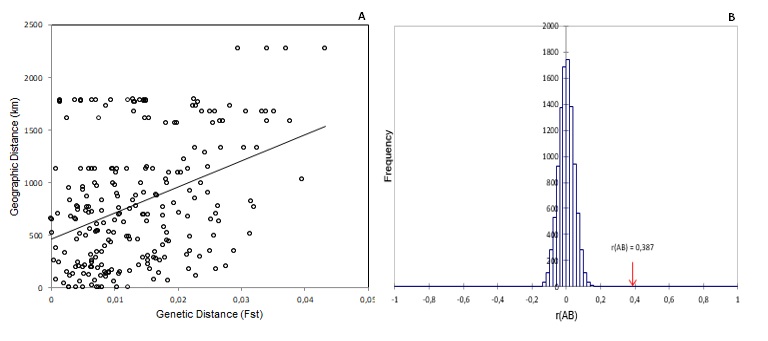

Supplement: Supplementary file 1 [file genes-16-00574-s001.zip › Figure_S5_SuppInfo.jpg]

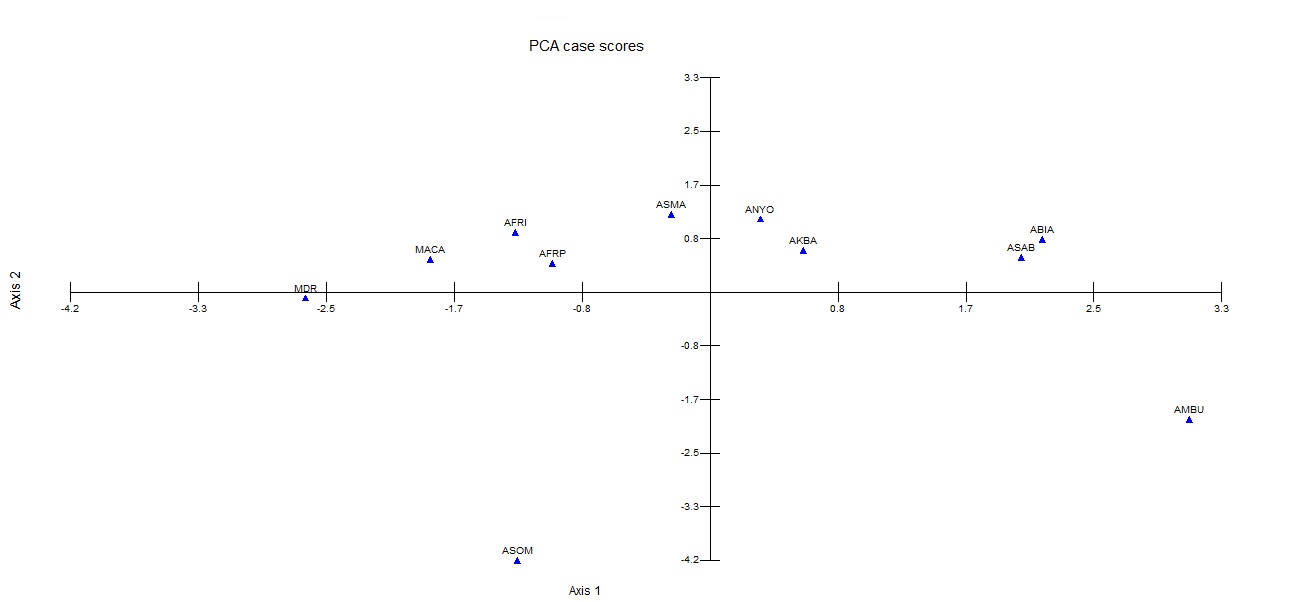

Supplement: Supplementary file 1 [file genes-16-00574-s001.zip › Figure_S6_SuppInfo.jpg]

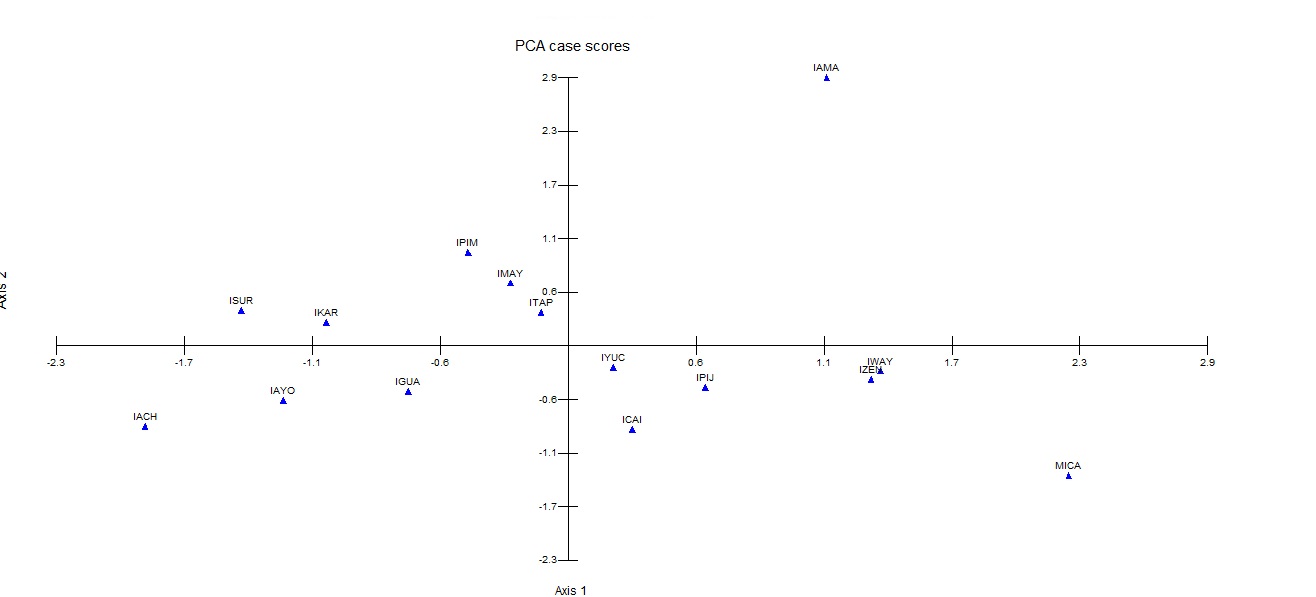

Supplement: Supplementary file 1 [file genes-16-00574-s001.zip › Figure_S7_SuppInfo.jpg]

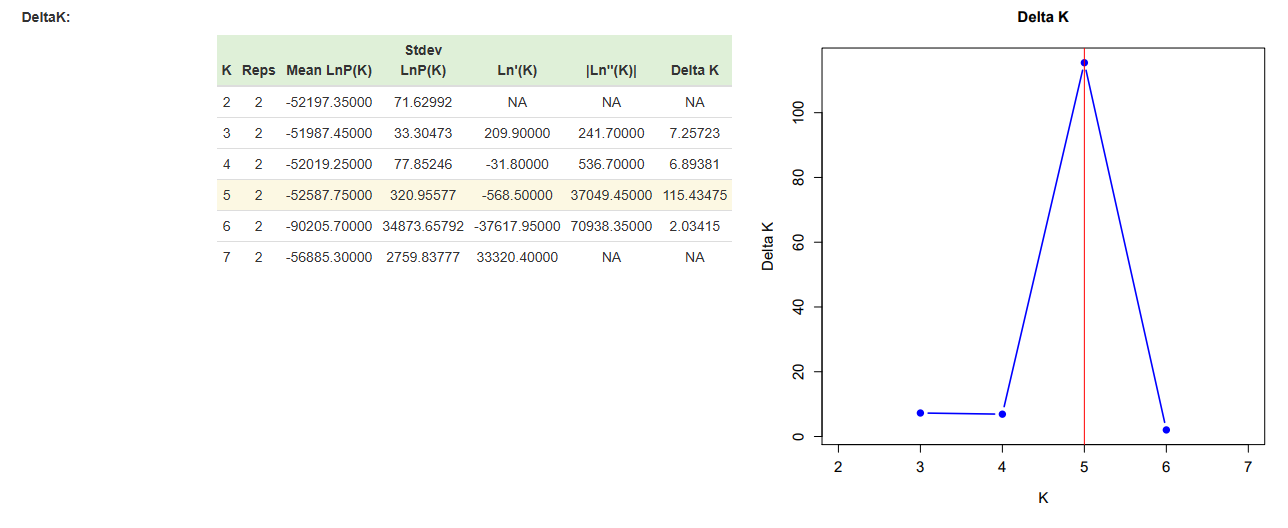

Supplement: Supplementary file 1 [file genes-16-00574-s001.zip › Figure_S8_suppInfo.png]
